# Vitamin D Dosing: Basic Principles and a Brief Algorithm (2021 Update)

**DOI:** 10.3390/nu13124415

**Published:** 2021-12-10

**Authors:** Andrius Bleizgys

**Affiliations:** Clinic of Internal Diseases, Family Medicine and Oncology, Faculty of Medicine, Vilnius University Santariškių 2, LT-08661 Vilnius, Lithuania; andrius.bleizgys@gmail.com

**Keywords:** vitamin D, calcidiol, supplementation, COVID-19

## Abstract

Nowadays, in modern societies, many people can be at high risk to have low vitamin D levels. Therefore, testing of serum 25-hydroxy-vitamin D (25OH-D) levels should be performed before prescribing them vitamin D supplementation. However, in some cases the 25OH-D level assessment is not available at the right moment, e.g., due to mandatory quarantine of COVID-19 outpatients. Therefore, such patients could be advised to start taking moderate vitamin D doses (e.g., 4000 IU/day for adults), and their 25-OH-D levels could be checked later. The proposed algorithm also comprises vitamin D dosing principles when baseline 25OH-D levels are known.

## 1. Introduction

The numbers of new COVID-19 cases and deaths from COVID-19 are increasing in many countries, despite the availability of different vaccines, more or less strict lockdowns or other state-level infection control measures, and various treatment options. It seems that there is a need for other effective tools for combating the COVID-19 disaster. Vitamin D (Vit. D) was suggested as one such tool [1]. Is it well known that Vit. D, in the form of calcitriol, has a pleotropic activity in human organism [2]. There is some evidence from clinical trials regarding the benefits of Vit. D for COVID-19 patients [3,4,5,6,7], as it was also stated in two recent reviews [8,9]. In the previous paper, the main mechanisms of Vit. D action in regard to COVID-19 infection were already discussed, and some suggestions for Vit. D dosing in the COVID-19 era were proposed [10]. Recently it was suggested that Vit. D might also act as an important cofactor of strengthening the activity of COVID-19 vaccines [11].

It is still debatable if checking the 25-hydroxyvitamin D levels (25OH-D-a marker of Vit. D status) and supplementing with Vit. D should be included in the COVID-19 prevention and treatment guidelines. Nevertheless, diagnosing, preventing, and treating low Vit. D status is still an issue in regard to both COVID-19 patients and the whole population, particularly keeping in mind the reduced accessibility to health care services during the COVID-19 pandemic. The current paper discusses the main Vit. D dosing principles, outlines the most important low Vit. D risk groups, and suggests a brief Vit. dose selection algorithm for clinical practice.

## 2. Is There a Need for New Guidelines/Algorithms?

During the past 10–15 years, different international and regional guidelines for low Vit. D status prevention and treatment were published (e.g., [12,13,14,15,16]). However, for several reasons, physicians might currently need some new kinds of recommendations for clinical practice regarding Vit. D status evaluation and Vit. D dosing.

1. Despite the available evidence of vitamin’s D important role for the human organism, including extra-skeletal health and the high prevalence of low Vit. D status in different regions of the world [17,18,19,20,21], many countries still do not have national, up-to-date, approved Vit. D guidelines. The same applies also to Lithuania, which has only the Rickets’ diagnosis and treatment guidelines approved in 2015. Moreover, in most countries, the potential beneficial role of Vit. D for COVID-19 prevention and treatment (i.e., Vit. D as an adjuvant) is still not accepted; consequently, no specialized relevant recommendations are developed. Paradoxically, it is the COVID-19 pandemic that inspired the author of the present article to start developing national Vit. D guidelines for Lithuania. Hopefully, the basic principles of those guidelines presented in the current paper could be an additional source for more specialized future recommendations both for Lithuania and for other countries.
2. Traditionally, any well-prepared Vit. D guidelines should reflect clinical practice and therefore must include the following domains: definition of risk groups for low vitamin D; principles of evaluation of Vit. D status by using laboratory measurements; and Vit. D dosing for prevention and treatment. However, the COVID-19 pandemic brought some challenges that aggravated our routine clinical practice. Firstly, due to reduced accessibility to health care facilities, mandatory isolation of some patients (due to diagnosed COVID-19 disease or due to close contact with a confirmed COVID-19 case), or a patient’s fear of getting SARS-CoV-2 during visits to a clinic or laboratory, it is not possible to perform the measurements of serum 25OH-D levels at the desired time. Therefore, the recent Vit. D status of many outpatients could remain unknown. Secondly, with the absence of data on recent 25OH-D level measurements, it might be difficult for physicians to make decisions regarding Vit. D dosing, particularly for low Vit. D risk group patients. We need an extended list of risk factors that might suggest the clinician to presume that certain patients could be put into a Vit. D risk group and, consequently, to suggest him/her higher Vit. D doses for supplementation. Finally, even disregarding the potentially beneficial direct Vit. D role on COVID-19 prevention and treatment, it is wise to remember that the problem of low Vit. D in society has not disappeared during the pandemic. Moreover, some people, due to various reasons during lockdowns, may have even higher risk to newly develop Vit. D insufficiency, leading to poorer skeletal and extra-skeletal health [10]. Patients having low 25OH-D levels might be considered as high-risk group for getting severe illness from COVID-19 [22].
3. In older Vit. D guidelines, there is almost no talk about the causes that could result in failure to achieve the desired levels of 25OH-D by supplementing Vit. D, and the suggested actions for physicians. In the present article, the author also tried, in part, to fulfil those gaps.
4. It is the Vit. D supplementation that modern guidelines should be mostly oriented to. Production of vitamin D3 in the skin is not a reliable source for repletion of low Vit. D status. Firstly, human skin is able to produce only limited amount of vitamin D3 that can enter the circulation [23,24]. Secondly, it is difficult to predict the effect of solar radiation in regard to vitamin D3 production and its influence on 25OH-D levels, since a large number of factors might affect vitamin D3 synthesis in the skin, e.g., skin type, patient age, time of the day, altitude, etc. [23,25,26]. Finally, in some countries, e.g., Lithuania, that are located at the middle latitudes, the intensity of solar radiation decreases significantly during the cold season, and the synthesis of vitamin D3 in the skin is almost absent during the period from October till March [27,28]. Food, unless fortified with Vit. D, usually cannot serve as a valuable source of this vitamin, too [27,29,30]. Therefore, this paper does not discuss recommendations on exposure to sunlight or influence of certain types of food for prevention or treatment of low Vit. D status.

## 3. Risk Factors for Low Vitamin D Status

There are a number of diseases and conditions associated with low Vit. D status. Illnesses definitely caused by inadequate Vit. D status comprise only a small part of the group of all risk factors for low Vit. D status. Many diseases, conditions, or drugs *per se* can impair vitamin’s D metabolism and/or increase the needs for this vitamin, thus contributing to development of low Vit. D status.

In addition, there are a lot of diseases and conditions where low Vit. D status can be considered only as an epiphenomenon. In other words, low Vit. D status itself does not necessarily have cause−consequence relationships with certain diseases or conditions, but it frequently accompanies them and could have common causes. In many cases, an unhealthy lifestyle can act as such a common cause, and low Vit. D status might serve as an indicator of that lifestyle [31,32,33,34].

It is worth to try to identify those risk factors, since some of them can be corrected (or prevented) and this may help to prevent and treat low Vit. D. In addition, COVID-19 disease and low Vit. D status share many risk factors, e.g., older age and obesity [10]. Therefore, considering together those risk factors (including low Vit. D status) can help to correctly evaluate the risk of severe COVID-19 and, in some cases, also advocate vaccination. On the other hand, confirmed symptomatic COVID-19 disease might be considered as risk factor for suspecting low Vit. D status.

For simplicity, risk factors for low Vit. D status can be divided into several groups (Table 1) [12,13,20,24,35,36,37,38,39,40,41]. Patients having one or several risk factors should be tested for their serum 25OH-D levels, since the analysis results helps in making better decisions regarding Vit. D supplementation [12,42,43].

## 4. Evaluation of Vitamin D Status

Vit. D status can be categorized by evaluating serum 25OH-D levels (Table 2) [10,12,24,35,40,44]. For many years, it has been argued that levels of 25OH-D should be at least 50 nmol/L, since this is sufficient to maintain good skeletal health in almost all individuals [20]. However, many experts claim that levels of 75 nmol/L and above are those sufficient to ensure normal skeletal and muscular structure and function [12,24,45,46]. There is growing evidence that minimum 100 nmol/L of 25OH-D levels are needed to reduce the risk of some cancers (e.g., colorectal), cardiovascular disease, infectious diseases, pathological pregnancies (e.g., preeclampsia, gestational diabetes, preterm birth), systemic connective tissue diseases, diabetes, and also COVID-19 [9,14,16,22,41,44,47,48,49,50]. An optimal (at least 100 nmol/L) levels of 25OH-D mean that Vit. D is sufficient for all systems in the human body, not only for bones [16]. Some authors speculated that a laboratory-determined concentration of 100 nmol/L indicates that the true serum 25OH-D levels of the individual are greater than 75 nmol/L [12]. In summary, 25OH-D levels of 75–150 nmol/L should be considered as “normal”. The term “low vitamin D status” used in the present paper comprises both Vit. D deficiency and Vit. D insufficiency, as defined in Table 2.

## 5. Vitamin D Dosing Principles

In order to simplify and not to overload the final brief algorithm of Vit. D dose selection, it is reasonable to present both prophylactic Vit. D doses and Vit. D doses for treatment separately. The aim of this article is not to discuss various advices on Vit. D dosing from different guidelines in depth; therefore, only summarized recommendations are presented.

Table 3 presents recommended Vit. D doses for prevention of low Vit. D status in different age groups [12,13,24,51]. In cases of Vit. D deficiency or insufficiency, therapeutic Vit. D doses should be prescribed according to both baseline 25OH-D levels and patient age (Table 4) [12,13,16,24,45,52,53,54,55].

For Vit. D risk group patients, particularly obese individuals or those having weight >90 kg as well as persons with malabsorption syndromes, Vit. D dose should be increased two-fold or sometimes even three-fold [12,16,24,35,40,46,48,56,57]. It is noteworthy that Vit. D doses up to 10,000 IU/d are considered safe for the vast majority of patients [44].

In patients with or at high risk of hypercalcemia, such as those with granulomatous disease, Vit. D dose should be adjusted individually by routine monitoring of calcemia, calciuria, 25OH-D, parathormone, and 1,25-dihydroxy-vitamin D levels [24]. For those patients, small Vit. D doses are suggested in order to maintain serum 25OH-D levels below 75 nmol/L [12].

In case of Vit. D overdosing, Vit. D supplementation should be stopped or at least halved and, if indicated, serum calcium levels should be measured. In case of Vit. D intoxication, which is an extremely rare condition when hypercalcemia occurs due to use of Vit. D supplements, Vit. D supplementation should be stopped, and the treatment of hypercalcemia should be applied (see [51,58,59] for details).

## 6. A Brief Algorithm for Vitamin D Dosing

**Figure 1:**
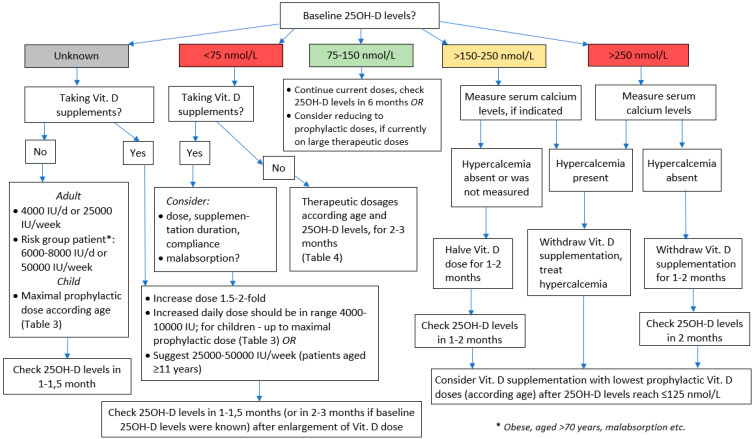
Brief algorithm for vitamin D dosing.

As mentioned above, even for Vit. D risk group patients, recent measurements of serum 25-OH-D are not always available. Therefore, presuming that the true levels of 25OH-D for many individuals could be below 75 nmol/L, it is reasonable to suggest starting vit. D supplementation with 4000 IU/d or an equivalent weekly dose. For patients definitely belonging to Vit. D risk group, except those with or at high risk of hypercalcemia (sarcoidosis, etc.), the initial Vit. D dose might be doubled (Figure 1). For patients that are already taking Vit. D supplements without having performed 25OH-D measurements and without physician’s advice before beginning of supplementation, it could be presumed that they might have low Vit. D status, i.e., they decided to start taking Vit. D on the grounds of their symptoms that potentially could have been caused by Vit. D insufficiency/deficiency. Therefore, it is reasonable to suggest doubling the Vit. D dose that they are currently using, but not exceeding the upper “safe” dose limits (10,000 IU/d).

In all those cases, 25OH-D levels should be checked at 1–1.5 months after the initiation of Vit. D supplementation or the enlargement of Vit. D dose, respectively. The span of 1–1.5 months was chosen for three reasons: (i) after that period, the mandatory isolation for the majority of patients is ended or other obstacles precluding their visit to the laboratory are solved; (ii) in some cases, it might help to evaluate the efficacy of supplementation (and to suspect, e.g., malabsorption), and (iii) to detect Vit. D overdose early enough [10,12,24,36,60].

The suggested original, brief or “working” algorithm presented in Figure 1 also comprises the main principles of Vit. D dose selection discussed earlier in this paper when baseline 25OH-D levels are known ([10,13,24,49]).

## 7. Dealing with Failure to Increase 25-Hydroxy-Vitamin D Levels

In cases where “adequate” Vit. D supplementation fails to improve Vit. D status, it is worth thinking over several things, such as:

1. The dose that was prescribed and the duration of supplementation. If the Vit. D dose could have been too small, it can be increased two-fold, and the next check of 25OH-D levels can be performed at 1.5–2 months after dose correction.
2. The compliance. Some patients prefer not to take large Vit. D doses even by physician prescription and, in fact, consume only small doses, for the fear of Vit. D overdose.
3. Possibility of unreported chronic diseases or use of certain drugs that could impair Vit. D metabolism. Some patients might be candidates to be examined for possible malabsorption syndrome, particularly in cases when 25OH-D levels did not increase significantly even after the supplementation with doubled dose. In some cases, e.g., for those with celiac disease, severe liver disease, or after bariatric surgery, calcidiol might be suggested, since it has better intestinal absorption than Vit. D and appears to be two to three times more effective in increasing serum 25OH-D levels than vitamin D3 [20], and this feature of calcidiol might be very important also in early stages of the COVID-19 disease, when low serum 25OH-D levels need to be increased as soon as possible [8,61].
4. Adequacy of calcium (Ca) and/or magnesium (Mg) intake. During the treatment of low Vit. D, supplementation with Mg (daily dose in the range 250–500 mg/d) is recommended, since Mg acts as a cofactor in many enzymes involved in Vit. D metabolism [44]. In addition, it is worth understanding that long-term decreased intake of Ca with food can, in turn, aggravate low Vit. D status because of compensatory hyperparathyroidism that increases the production of calcitriol in the kidney from 25OH-D, and this consequently contributes to diminishing serum 25OH-D levels. Therefore, with adequate Ca intake (including Ca from supplementation, if necessary), a better response to Vit. D preparations can be expected [39,62]. The recommended daily intake of Ca for adults is ~1000–1200 mg; more data about Ca inadequacy can be found elsewhere [63].

## 8. Conclusions

Due to various risk factors, many COVID-19 and other patients are at high risk to develop low vitamin D status. If possible, it is reasonable to check their serum 25-hydroxy-vitamin D levels, and only after that, an appropriate dose of vitamin D supplements should be suggested. In case 25-hydroxy-vitamin D measurements are not available, taking moderate vitamin D doses (e.g., at least 4000 IU/d) could be advised for at least 1–1.5 months, presuming that this supplementation can contribute to reaching adequate vitamin D status and can help to maintain better overall health status—both skeletal and non-skeletal—until serum 25-hydroxy-vitamin D testing will be accessible for the current patient. If low vitamin D status is confirmed, an appropriate, large enough vitamin D dose must be suggested for supplementation, and the next check of serum 25-hydroxy-vitamin D levels should be advised after the treatment in order to evaluate treatment results and choose the right tactic regarding further supplementation.

## Figures and Tables

**Table 1:** Risk factors for low Vit. D status.

Groups of Risk Factors	Examples: Diseases, Conditions, Lifestyle Features
Musculoskeletal disorders	Rickets, osteoporosis, osteopenia, “bone pains”, muscle pain, myopathy, myodystrophy, recurrent (“low energy”) bone fractures, recurrent falls, bone deformities
Endocrine and metabolic diseases/conditions	Diabetes mellitus (type I and II), metabolic syndrome, obesity, overweight, hypo- and hyperparathyroidism, hypo- and hyperthyroidism, hypocalcemia, calciuria, phosphatemia, hypo- and hyperphosphatasia, phosphaturia, dyslipidemias
Increased demand for physiological reasons	Childhood, adolescence, pregnancy, breastfeeding
Malabsorption syndromes	Pancreatic exocrine insufficiency (old age, pancreatitis, type II diabetes, etc.), inflammatory bowel disease (Crohn’s disease, ulcerative colitis), cystic fibrosis, lactose intolerance, celiac disease, bariatric surgery
Diseases of the liver and bile ducts	Hepatic insufficiency, cirrhosis of the liver, cholestasis, hepatosteatosis
Kidney diseases	Renal insufficiency, chronic kidney disease (especially stages III–V), nephrotic syndrome
Respiratory diseases	Bronchial asthma, chronic obstructive pulmonary disease
Infectious diseases	Tuberculosis, recurrent respiratory infections
Systemic connective tissue diseases	Rheumatoid arthritis, systemic lupus erythematosus, dermatomyositis, fibromyalgia
Skin diseases	Atopic dermatitis, psoriasis
Diseases of the nervous system	Multiple sclerosis, Parkinson’s disease, dementia, cerebral palsy, autism
Decreased production of vitamin D3 in the skin	Older age (especially >70 years) Active protection against sun exposure (sunscreens, etc.) Cultural features (usual full-body clothing) Rare outdoor activities (work and leisure predominantly indoors; living in a care home) Increased air pollution (living in a city) Winter season (at medium latitudes) Dark-skinned (especially Africans)
Nutritional features	Veganism and other types of vegetarianism Allergy to cow’s milk Low-fat diet Insufficient magnesium intake Insufficient calcium intake
Long-term use of drugs	Antiepileptic drugs (e.g., valproate, phenytoin); antiretroviral drugs; glucocorticoids; systemic antifungal drugs; rifampin; bile acid sequestrants (cholestyramine); lipase inhibitors (orlistat)
Malignant neoplasms	Colon cancer, lymphatic system and blood cancers, breast cancer, ovarian cancer, prostate cancer
Granulomatous diseases	Sarcoidosis, histoplasmosis, coccidiomycosis, berylliosis
Mental illnesses	Depression, schizophrenia, anorexia nervosa
Cardiovascular diseases	Arterial hypertension, ischemic heart disease, heart failure
Others	Chronic fatigue syndrome Inpatient treatment (especially in the resuscitation and intensive care unit) Awaiting organ transplantation and post-transplant

**Table 2:** Vit. D status categories by 25OH-D levels.

Category	25OH-D Levels, nmol/L
Severe deficiency	<25
Moderate deficiency	25–<50
Insufficiency	50–<75
Sufficiency	75–<100
Optimal levels (optimal levels in tissues/cells)	100–<150
Increased levels	150–<250
Overdose	≥250
Intoxication *	≥375

* Intoxication category also includes lower 25OH-D levels, if hypercalcemia is caused by vitamin D supplements. 25OH-D–serum 25-hydroxy-vitamin D levels.

**Table 3:** Vitamin D prophylactic doses.

Patient Age	Recommended Daily Dose (IU/d)	Recommended Intermittent Dose	Upper Tolerable Daily Dose (IU)
Infants < 6 months	400–600	–	1000
Infants 6–<12 months	600–800	–	1000
Children 1–10 yrs.	600–1000	–	2000
Teens 11–<18 yrs.	800–2000	25,000 IU in 5–2 weeks	4000
Adults 18–<75 yrs.	1000–2000	25,000 IU in 4–2 weeks	4000
Adults ≥ 75 yrs.	2000–4000	25,000 IU in 2–1 weeks	4000

IU–international units.

**Table 4:** Vitamin D therapeutic doses.

Patient Age	Recommended Daily Dose and Duration	Recommended Intermittent Dose and Duration
**25OH-D Levels < 25 nmol/L**		
Infants < 1 month	1000 IU/d 3 months	–
Infants 1–<12 months	2000 IU/d 3 months	–
Children 1–<11 yrs.	3000–6000 IU/d 3 months	–
Children 11–<18 yrs.	6000 IU/d 3 months	50,000 IU/week 1.5–2 months
Adults	6000 IU/d 3 months	50,000 IU/week 2 months
**25OH-D Levels 25–<75 nmol/L**		
Infants < 1 month	Previously taking vit. D supplements: increase dose 1.5–2-fold No previous vit. D supplementation: largest prophylactic dose for age group (Table 3) Duration 2 months	–
Children 1–10 yrs.		–
Children 11–<18 yrs.		25,000 IU/week 2 months
Adults	Previously taking vit. D supplements: increase dose 1.5–2-fold No previous vit. D supplementation: largest prophylactic dose for age group (Table 3) Duration 2–3 months	50,000 IU/week 2 months

IU–international units; 25OH-D–serum 25-hydroxy-vitamin D levels.

## Data Availability

Not applicable.
